# Preliminary Observations and Impact in Japan of the Tsunami Caused by the Tonga Volcanic Eruption on January 15, 2022

**DOI:** 10.1007/s00024-022-03058-0

**Published:** 2022-06-04

**Authors:** Fumihiko Imamura, Anawat Suppasri, Taro Arikawa, Shunichi Koshimura, Kenji Satake, Yuichiro Tanioka

**Affiliations:** 1 International Research Institute of Disaster Science (IRIDeS), Tohoku University, 468-1 Aoba, Aramaki, Aoba-ku, Sendai, Miyagi Japan; 2 Faculty of Science and Engineering, Chuo University, 1-13-27 Kasuga, Bunkyo-ku, Tokyo, Japan; 3 Earthquake Research Institute, The University of Tokyo, 1-1-1 Yayoi, Bunkyo-ku, Tokyo, Japan; 4 Institute of Seismology and Volcanology, Hokkaido University, Kita-10 Nishi-8, Kita-ku, Sapporo, Japan

**Keywords:** 2022 Hunga Tonga-Hunga Ha’apai eruption and tsunami, tsunami observation, tsunami warning, tsunami evacuation, tsunami damage

## Abstract

The tsunami caused by the Tonga submarine volcanic eruption that occurred at 13:15 Japan Time (JST) on January 15, 2022, exposed a blind spot in Japan’s tsunami monitoring and warning system, which was established in 1952 for local tsunamis and expanded to distant tsunamis after the 1960 Chile tsunami. This paper summarizes how the warning system responded to the unprecedented tsunami, the actual evacuation process, and the damage it caused in Japan. Initially, the tsunami from the volcanic eruption was expected to arrive at approximately midnight with amplitudes of less than 20 cm. However, a series of short waves arrived at approximately 21:00, a few hours earlier than expected. The early arrival of these sea waves coincided with a rapid increase in atmospheric pressure; then, the short-period component was predominant, and the wave height was amplified while forming wave groups. After a 1.2 m tsunami was observed in Amami City in southern Japan at 23:55 JST, the Japan Meteorological Agency issued a tsunami warning/advisory. The tsunami continued, and all advisories were cleared at 14:00 JST on January 16. Information about this tsunami and the response to it are summarized here, including the characteristics and issues of the actual tsunami evacuation situation in each region. There were no casualties, but the issues that emerged included difficulty evacuating on a winter night and traffic congestion due to evacuation by car and under the conditions of the COVID-19 pandemic. In the coastal area, damage to fishing boats and aquaculture facilities was reported due to the flow of the tsunami. In addition, damage to aquaculture facilities, including those producing oysters, scallops, seaweed and other marine products, decreased the supply of marine products, and the economic impact is likely to increase in the future.

## Observations and Characteristics of Atmospheric Pressure and Tsunami in Japan

### Submarine Eruption and Tsunami Occurrence

On January 15, 2022, at 13:14 JST (4:14 UTC), a large-scale eruption occurred at the “Hunga Tonga-Hunga Ha’apai” submarine volcano off the coast of Tonga in the South Pacific Ocean. Eruptions have occurred intermittently on this volcanic island since December 2021. This time, the eruption size was reportedly close to that of Mt. Pinatubo in the Philippines in 1991, which is considered to be the largest in the twentieth century, with a volcanic eruption index (VEI) of 6. In the event of 2022, a series of large-scale submarine volcano eruptions caused tsunamis in many countries along the Pacific coast. There was a fragmentary damage report from the New Zealand government (Reuters, 2022) that a tsunami of more than 15 m had struck Tonga and caused damage around that area, but the details were unknown. Tsunamis were observed not only around Tonga but also in the Pacific Ocean, the Caribbean Sea, and the Mediterranean Sea (Reuters, 2022). There was also a report that the tsunami had exceeded 1.2 m in Japan (JMA, 2022) and two deaths occurred in a car in Peru, where the observed tsunami was 2 m (The Guardian, 2022). Furthermore, according to the US National Oceanic and Atmospheric Administration (NOAA), tsunamis of 1 m or more were observed in western California and Alaska (CNN, 2022).

### Observation and Warning in Japan

**Fig. 1:**
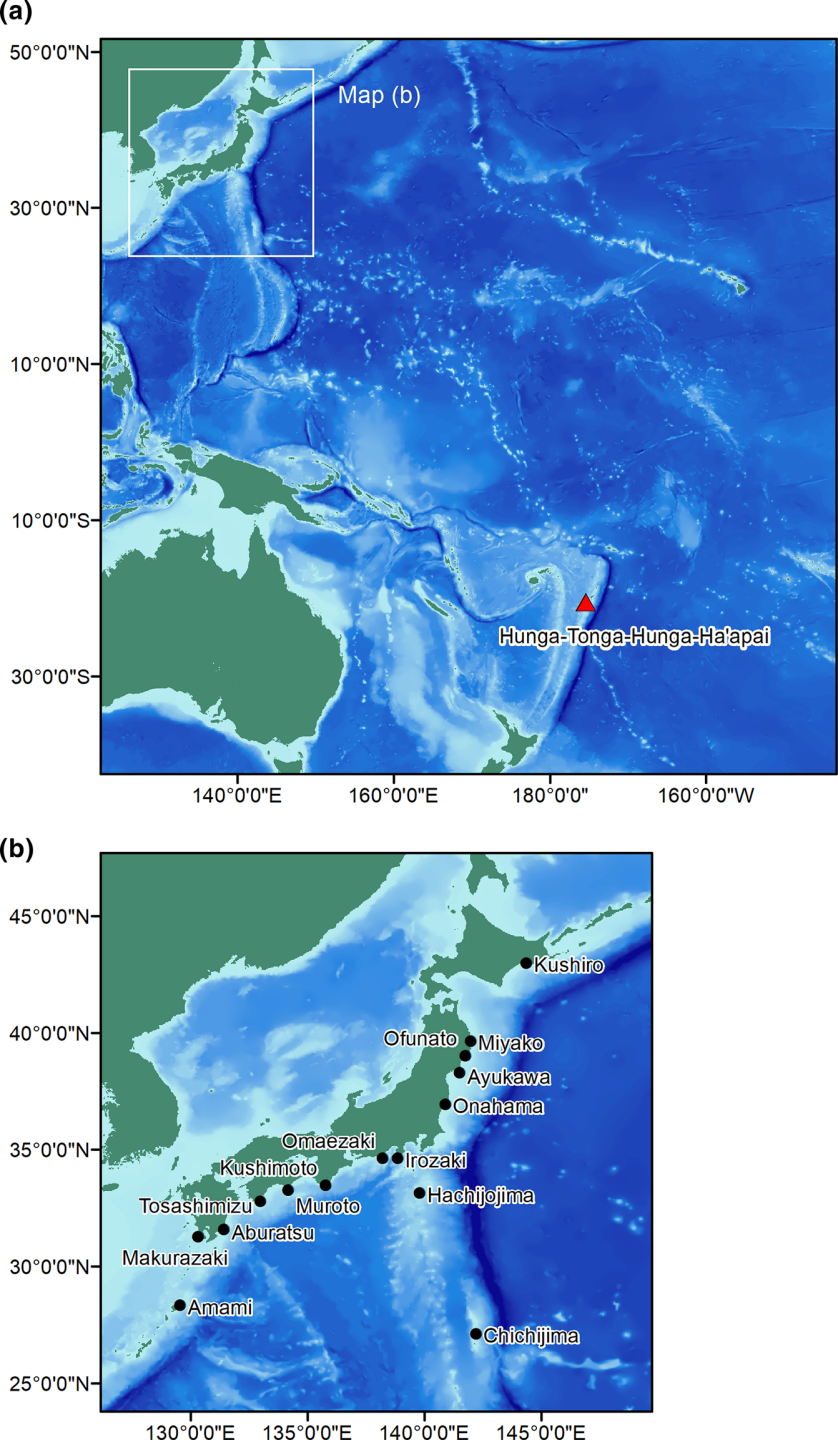
Geographical relationship Hunga Tonga-Hunga Ha'apai and Japan

**Fig. 2:**
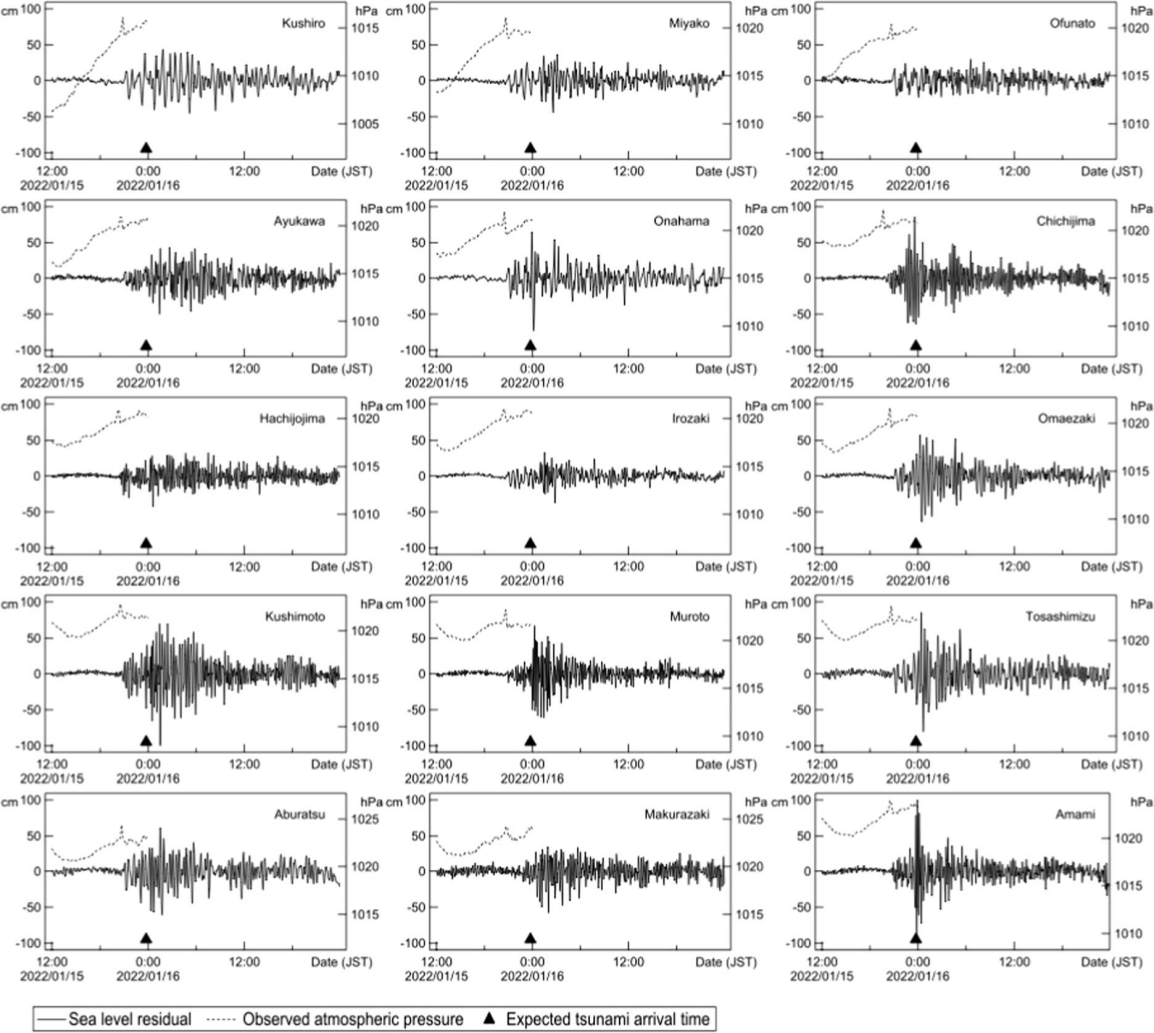
Record of atmospheric pressure and tsunamis (Detided). Atmospheric pressure was observed by the Japan Meteorological Agency (JMA), Tide level was measured by both Japan Coast Guard and JMA

Volcanic eruption and tsunami information was issued by the Pacific Tsunami Warning Center, but it was at a cautionary/advisory level. The Japan Meteorological Agency (JMA) reported that there was no significant tide-level rise on islands along the way to Japan, such as Tuvalu, Kiribati, and Micronesia. Therefore, the level in Japan was expected to be less than 0.2 m, which would have been below the tsunami advisory level (0.2 m is the criterion for marine damage caused by tsunamis in Japan). The JMA predicted that the arrival in Japan would be approximately 10 h after the eruption, that is, 23:15 JST or later. However, the tide level began to rise at approximately 20:00 JST, and 1.2 m was observed in Amami City, Kagoshima Prefecture, at 23:55 JST, as shown in Figs. 1, 2 and Table 1. The actual tsunami was observed 2–3 h earlier than the estimated arrival time, at approximately the same time as the atmospheric wave (Asahi, 2022a; The Japan Times, 2022).

**Table 1:** Time table of the situation of the volcanic eruption and tsunami warnings in Japan and the Pacific on January 15, 2022 (time in JST)

Time (JST)	Observation/information of eruption and tsunami	Japan Meteorological Agency	PTWC
15 January 13:14	Large-scale volcanic eruption in Tonga		
14:31	0.6 m tsunami observed in Samoa (US)		
14:48			Issue of tsunami advisory for American Samoa
15:36			Issue of tsunami warning for American Samoa
16:39			Warning cancelled for American Samoa
17:06			No damage expected in Hawaii
18:00		Information on volcanic eruption in Tonga	
18:29	0.1 m tsunami observed in Hawaii		
19:01		Slight sea-level fluctuations expected	
19:43			Issue of tsunami advisory for Hawaii
19:58	1st tsunami observed in Chichijima Island		
20:13	Atmospheric wave observed in Japan		
20:20	1st tsunami observed in Katsuura City, Kanto region		
21:53			Issue of tsunami advisory for west of US and Aleutians
23:55	1.2 m tsunami observed in Amami City, Kyushu region		
16 January 0:15		Issue of tsunami warning for Amami and Tokara islands Issue of tsunami advisory in the Pacific coastal areas from the eastern part of the Pacific coast of Hokkaido to the Miyakojima and Yaeyama regions	
2:26	1.1 m tsunami observed in Kuji City, Tohoku region		
2:52			Advisory cancelled for Hawaii
2:54		Issue of tsunami warning for Iwate Prefecture	
4:07		Issue of tsunami advisory for western Nagasaki Prefecture and western Kagoshima Prefecture	
7:30		Tsunami warning was reduced to a tsunami advisory for Amami and Tokara islands	
9:31			Advisory cancelled for US Alaska and Aleutians, Washington, Oregon
11:20		Tsunami warning was reduced to a tsunami advisory for Iwate Prefecture	
14:00		All of Advisories canceled in Japan	
17:26			Advisory cancelled for California

### Characteristics of the Tsunami Observed in Areas Far from Tonga

Figure 2 shows a comparison of atmospheric pressure changes and tsunami observation records at 15 representative locations in various parts of Japan. In Japan, 188 tide gauges have been installed nationwide, and on this occasion, tsunamis were observed at 28 locations on the Pacific side. The tide level began to change after sudden changes in atmospheric pressure were observed in various parts of Japan. Notably, the tsunami started to reach Japan approximately 3 h earlier than the expected arrival time in the form of normal gravity waves. First, the tide level fluctuated as a tsunami with an increase in atmospheric pressure of approximately 2 hPa; then, the first peak occurred at approximately 23:00 JST to 24:00 JST, while the short-period component gradually amplified. The time of after 23:00 JST corresponded to the forecast time of the tsunami arrival as normal gravity waves. After that, the tsunami repeatedly vibrated in a short cycle of several minutes to 10 min while drawing an envelope. The time interval of sea-level rise and fall was characterized by the predominance of short components not found in ordinary distant tsunamis.

The type of tsunami that occurred after the 1883 eruption of Krakatau in Indonesia (Choi et al., 2003; Symons, 1888; Yokoyama, 1981) has been observed worldwide and is characterized by the predominance of short-period components similar to this one; in these cases, the tsunami arrival time is estimated by normal gravity waves (Pelinovsky et al., 2005). This feature is caused by the rapid rise in pressure (shock wave) due to the eruption as air-sea waves, as described in Harkrider and Press (1967) and Press and Harkrider (1966). In the 2022 instance, the air pressure change due to the eruption was considered to cause the sea-level fluctuation, which was then amplified in the process of being transmitted to Japan.

The tsunami that hit the area around Tonga was reportedly 15 m high, and it is possible that this tsunami, which had a large inundation range, was caused by volcanic eruption processes, such as pyroclastic flows or caldera depressions. However, the detailed mechanism and situation are still unknown. The tsunami observed in the archipelago of Micronesia, such as New Caledonia, which is located between Tonga and Japan, was reportedly 0.1–0.3 m, which is much smaller than the 1.2 m observed in Japan. It is possible that the main components were overlooked because of the relationship between the location of tide stations and the direction of the main tsunami energy heading toward Japan. The tsunami as a gravitational wave due to the activity around the Tonga eruption may have had different effects on various places (Choi et al., 2003; Kawamata et al., 1992; Nomanbhoy & Satake, 1995), but it has not yet been quantitatively evaluated. In addition to the abovementioned air vibration tsunami, the excitation in the propagation process of troughs and ridges on the way and the tsunami as a gravity wave generated around Tonga are thought to have also reached Japan, although the scale might have been small. It is necessary to analyze the situation in Japan in the future by paying attention to the following viewpoints when comparing observation records and numerical analysis results:

1. With the eruption of Tonga Hunga at 13:15 JST, the atmospheric pressure change propagated as a Lamb wave at the speed of sound, approximately 310 m/s. It was observed at approximately 19:00 JST in Chichijima Island and approximately 20:00 JST in Honshu (the main island) with an amplitude of approximately 2 hPa. Honshu is the largest island shown in Fig. 1 and includes Miyako, Ofunato, Ayukawa, Onahama, Irozaki, Omaezaki, and Kushimoto as observation points.
2. At about the same time, sea-level fluctuations began to be recorded on the deep-sea floor and along the coast. Unlike atmospheric Lamb waves, they continued for a long time because when the Lamb waves propagated in the Pacific Ocean over a long distance, the propagation velocity was close to the propagation velocity of the tsunami, which was caused by the coupling of the atmospheric and ocean fluctuations. It is therefore possible that “tsunamis” arrived in succession. This phenomenon is similar to that measured during the explosion of Krakatau volcano (Harkrider & Press, 1967).
3. The tide gauge records along the coast indicate that there were many points where the amplitude suddenly increased around the expected arrival time of the tsunami from Tonga Hunga at 22:30 JST in Chichijima Island and after 23:30 JST in Honshu. The tsunami component of the volcanic eruption may have been added.

## Tsunami Forecast, Evacuation Order and Actual Situation

### Tsunami Warning/Advisory and Evacuation

As shown in Table 1, according to the JMA (Japan Meteorological Agency, 2022), a tsunami warning was issued to the Amami Islands and Tokara Islands at 0:15 JST (January 15, 15:15 UTC) on January 16. Tsunami advisories were issued in the Pacific coastal areas from the eastern part of the Pacific coast of Hokkaido to the Miyakojima and Yaeyama regions. Then, at 2:54 JST on January 16, the tsunami warning was also issued to Iwate Prefecture, and at 4:07 JST on the same day, new tsunami advisories were announced for western Nagasaki Prefecture and western Kagoshima Prefecture. The tsunami warning was reduced to a tsunami advisory at 7:30 JST on January 16 for the Amami Islands and Tokara Islands and at 11:20 JST on the same day for Iwate Prefecture. All tsunami advisories in Japan were canceled at 14:00 JST. In this way, the tsunami advisory was shifted to a tsunami warning according to the observation information. Additionally, there was a problem of sequentially obtaining and responding to this information.

Based on this information, an evacuation order was issued, for example, 3 min later in Amami City, Amami Oshima Island, where the warning was issued, and 10 min later in Setouchi Town. For the Amami Islands, Tokara Islands, and Iwate Prefecture, where warnings were issued, the evacuation status was investigated based on materials published on the Internet. In the coastal areas of Iwate Prefecture, While individual media outlets have different figures [e.g., approximately 10% (Asahi, 2022b) and a few percent (The Mainichi, 2022)], it is understood that the evacuation rate (as a percentage of the municipality’s population) is approximately 10%. It is possible that evacuation to places other than evacuation centers or vertical evacuation (shelter in place) occurred on an individual level. Therefore, it is necessary in the future to investigate in detail why such differences occurred. In the next section, detailed information and an evacuation status report are provided for Amami Oshima Island.

### Evacuation Situation in Amami Oshima

**Fig. 3:**
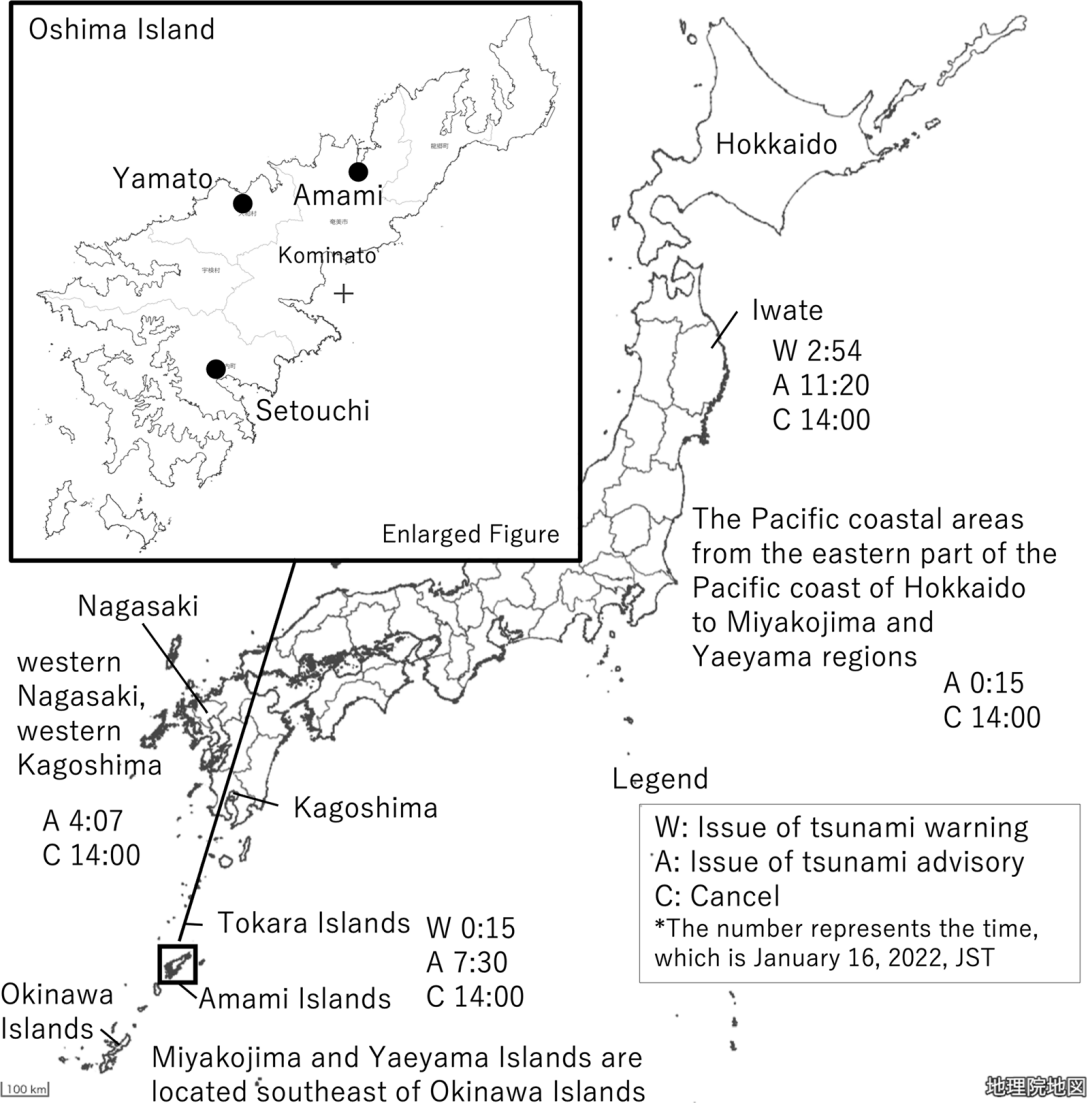
Area in Amami Oshima Island, Kagoshima Prefecture (area targeted for evacuation survey)

Interviews were conducted in Amami City, Setouchi Town, Yamato Village, and Kominato District of Amami Oshima Kagoshima Prefecture (Fig. 3), and the results indicated that a considerable number of people evacuated immediately after the warning was issued. The major reasons were experience of the 2011 Great East Japan Earthquake (Asahi, 2022b) and the memory of flooding during the 1960 Chile tsunami. Another factor was that the embankment is low, the sea can be seen, and the land is flat, so people recognized that the situation would be dangerous once inundation occurred.

In terms of the evacuation ratio, approximately 960 out of 1400 people in Yamato Village evacuated, and most of those in Setouchi Town evacuated, including vertical evacuation to the 2nd and 3rd floors. In Amami City, government staff commented that the line of cars trapped in traffic started at 24:18 JST, 3 min after the warning was issued. This is proof that the evacuation started not only because of the evacuation order of the local government but also because of the JMA tsunami warning. We also found that quite a few people returned home after the alarm was cancelled.

In the Kominato area, evacuation drills could not be conducted for the previous two years due to the COVID-19 pandemic, and although evacuation sites were not properly designated, people individually chose to evacuate to high ground such as mountains. In addition, evacuation was instigated by calling out to family members and acquaintances, showing that the initiative of evacuees is important.

Evacuation using cars was a major problem in almost all areas. There was no noticeable traffic jam in Yamato Village, but there was a traffic jam that did not move for approximately one hour in Amami City. The reasons may be that many elderly people regularly use cars, people were going to the mountains and evacuating with children and others requiring assistance, and they were evacuating at night. Since roads may be damaged during an earthquake, it is necessary for local residents to discuss how to manage evacuations by car.

In Yamato Village, there was a facility only for people requiring assistance on the seaside, and there were few vehicles to move people requiring assistance to higher ground, so it took approximately 1 h and 30 min from the time of the warning for the evacuation to be completed. This problem may occur for many Japanese local governments in the future, and we must consider a solution. Furthermore, it is important to prepare for disasters on a daily basis, for example, by improving toilet facilities and taking seasonal measures such as procedures to deal with cold. Under such circumstances, the characteristics of an island often appeared; for example, communication between neighbors occurred on a daily basis, and mutual help was relatively available even in this tsunami. “It is important to continue to improve communication in the village and deal with various problems,” said a local government official.

### Issues for Evacuation

**Photo 1:**
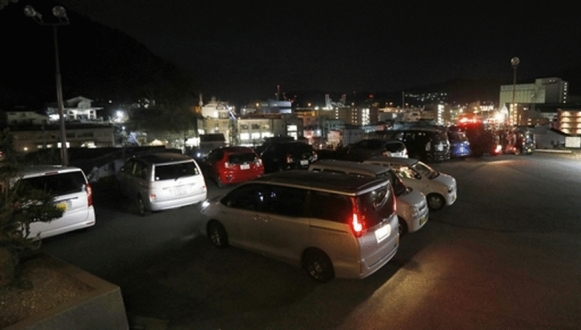
Cars gathered on a hill (Kamaishi City, Iwate Prefecture). A tsunami warning was issued, and the cars of evacuees gathered at a temple on a hill = 3:52 am on January 16 [Provided by Kyodo News]

**Photo 2:**
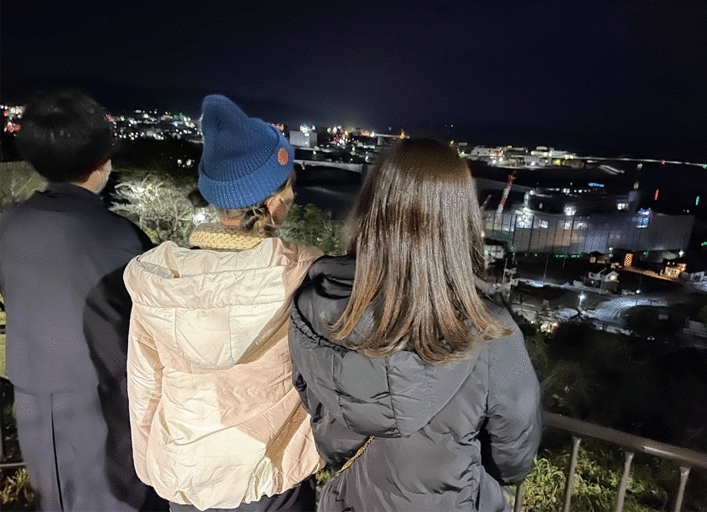
Evacuees gathered on a hill (Hiyoriyama, Ishinomaki City affected by the 2011 Great East Japan Earthquake tsunami) [Provided by Sanriku Kahoku Shimpo]

Regarding the use of cars in a tsunami evacuation, many people reportedly evacuated by car not only in Amami Oshima Island but also in Ofunato and Kamaishi City, Iwate Prefecture (Photo 1, Kyodo, 2022); thus, it is possible that car evacuation has become usual. Travel by car occurs daily in an aging society, and cars are often indispensable as a means of transportation. Therefore, it is necessary to consider realistic measures according to the state of society. Evacuation at night hinders evacuation behavior. It is also possible that evacuation by car may be necessary after drinking alcohol with a meal, even though drunk driving laws are very strict in Japan. In addition, it was difficult to evacuate uphill at night due to the cold winter weather (Photo 2). In response to hoax information such as that posted on social network sites (SNS) (for example, a video of another tsunami shown as a video of the current tsunami), it is important that information be provided by reliable resources. It is currently difficult to deal with this problem, but it is important to build a mechanism that allows users to instantly judge the reliability of information.

Finally, as an issue in the evacuation fact-finding survey in Amami Oshima Island, it was difficult to identify who was where when evacuees voluntarily evacuated to higher ground, which may make survival in the event of a major disaster more difficult, as it seemed that collecting the information would be time consuming. In response to this problem, it is necessary to develop a mechanism to collect the behavioral information of all evacuees and monitor their behavior as much as possible, but how can such monitoring be balanced with privacy issues? This task will be difficult. Additionally, at Sakihama Port in Kochi Prefecture, the disaster prevention administration radio did not sound, and many fishermen went to see the state of the port. Since the tsunami hit at night, no offshore damage was reported. It is necessary to grasp the details of actual damage and especially consider the rules for evacuation of fishing boats when a crisis occurs during the day in areas other than those affected by the 2011 Great East Japan Earthquake tsunami.

## Fishery Damage Along the Coast

### Overview

**Photo 3:**
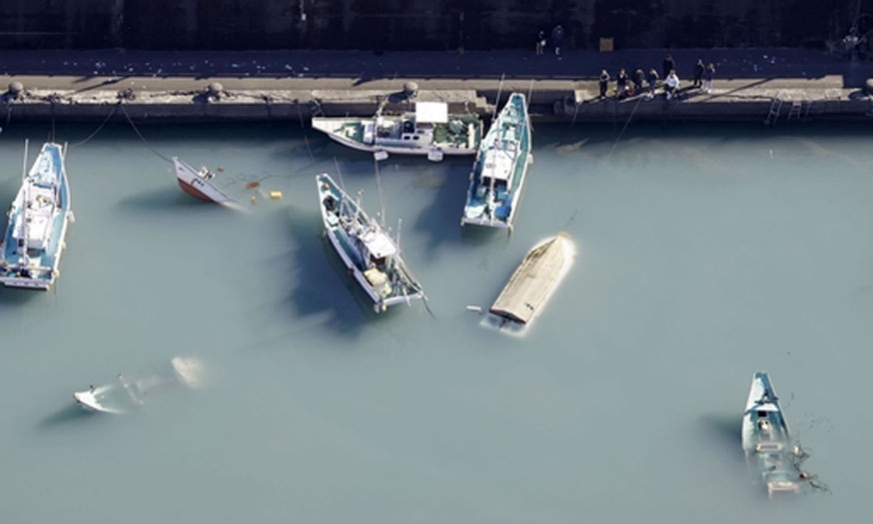
Capsized fishing boat 3 (Muroto City, Kochi Prefecture). Port of Muroto City, Kochi Prefecture, where multiple ships capsized or sank = 10:24 JST on January 16 (from Kyodo News helicopter) [Provided by Kyodo News]

This tsunami had no runup or overflow of riverbanks and was not on a scale that would cause enormous damage (Kyodo, 2022), but damage and effects in the sea area were reported (Photo 3). The tsunami occurred at the time to harvest oysters, scallops, and early-picked wakame seaweed, and there is concern that the damage will continue to spread. In Japan, fisheries have been damaged in distant tsunamis, including the 1960 Chile tsunami, the 2010 Chile tsunami (Kato et al., 2010), and the 2011 Great East Japan Earthquake tsunami. As we have learned from damage caused by past tsunamis with long wave periods, damage begins to occur to fishing boats when the wave height is 1 m or the flow velocity is 1 m/s or more (Muhari et al., 2015; Suppasri et al., 2014) and to aquaculture facilities when the flow velocity is 1 m/s regardless of the maximum water level (Suppasri et al., 2018). However, no such damage has been caused by non-seismic tsunamis (short-period tsunamis).

### Damage Situation

**Fig. 4:**
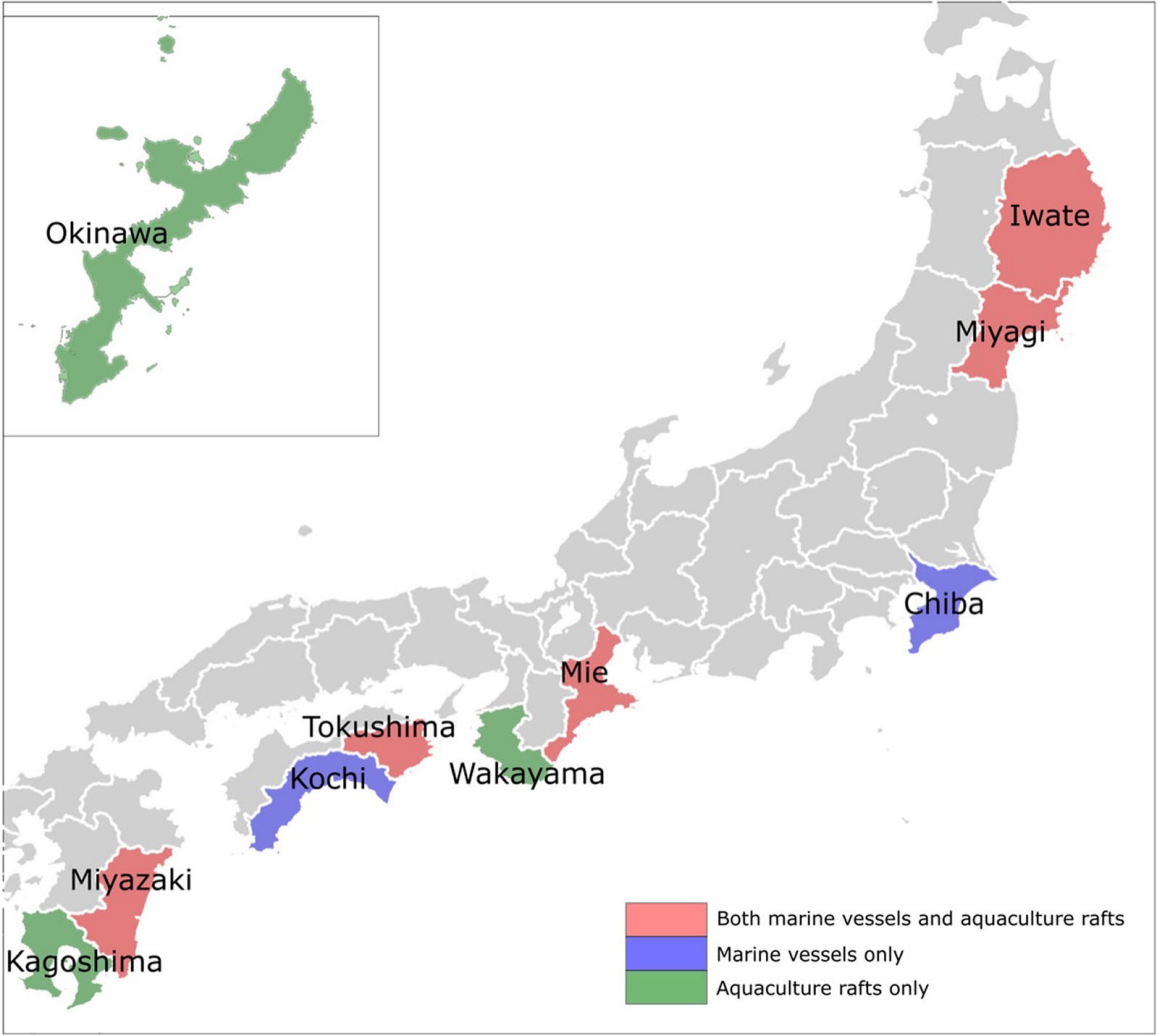
Major fishery damage along the coast of Japan

Since the tsunami hit Japan at night, the actual damage was known beginning on January 16, and much news was reported on January 17. Although the damage to fishing boats can be quickly collected because they are on the sea surface, it took time to collect damage reports for aquaculture facilities because they are underwater, and the work could be started only after the tsunami warning/advisory was canceled. According to the Ministry of Agriculture, Forestry and Fisheries, as of 14:00 JST on January 20, damage was reported to 37 vessels in 5 prefectures, including Kochi Prefecture, owing to fishing boats in port being overturned and sunk, and damage was reported to 7 fishing gear and aquaculture facilities in Tokushima Prefecture (Fig. 4). Regarding farmed fish, there were four reports in three prefectures, including the death of yellowtail artificial seedlings in Kagoshima Prefecture due to threading with net cages. However, the amount of damage is not yet fully understood. According to our interviews with fishermen, there were two major causes of damage to aquaculture facilities.

1. Location of aquaculture facilities: Basically, the locations of these facilities vary by several meters to several tens of meters depending on the area, the types of marine products or the navigational conditions. The interviewees pointed out that the damage from this tsunami was mostly in areas at shallow depths (i.e., Matsushima Bay). In such areas, weak structures are normally used due to comparatively calm wave conditions.
2. Installation method: The strength of aquaculture facilities varies widely depending on the seabed type (i.e., sand or mud) or whether pile or anchor is used.

For example, Matsushima Bay, which was damaged in Miyagi Prefecture, is in a shallow sea area and is a wooden frame type (using piles). Currently, it is still not clear whether the damage was only because of the structure or was also related to other physical aspects of the tsunamis, such as shoaling and resonance. Therefore, in future surveys and studies, it is necessary to consider not only the characteristics of the tsunami, such as wave height, flow velocity, and wave period, but also the seabed type and installation method.

### Future Issues and Countermeasures

According to satellite images, approximately 50 fishing boats are normally moored at Sakihama Port, Muroto City, Kochi Prefecture, which suffered the most damage. Since 10 ships were damaged, the damage ratio can be estimated to be approximately 20%. Few fishing boats were damaged when the 2010 Chile tsunami struck, which indicates that short-period tsunamis are more likely to damage fishing boats; thus, it is necessary to consider measures such as mooring methods in the future. In a long-period tsunami, a rope several tens of meters long will be in a tense state, and the mooring rope and anchor will not be able to resist the fluid force acting on the boat or aquaculture facility and will moved or break. This means that the drift of the boat or aquaculture facility is not necessarily related to the water level, but the influence of flow velocity is significant. On the other hand, flow velocity on the vertical axis is fast in a short-period tsunami, but the rise in water level has little effect on damage. Hence, the damage situation is limited, as in this case, to instances of ropes that might become entangled. Nevertheless, such damage is difficult to prevent considering the cost of the structure of the facility or of building offshore coastal structures. It seems more effective to implement economic policies such as insurance and sales support for regional products to mitigate the damage. Currently, Iwate, Miyagi and Mie Prefectures are beginning to consider ways to support aquaculture companies.

## Summary

Even in Japan, which is 8500 km away from the Tonga submarine volcanic eruption, tsunamis were observed along various parts of the Pacific coast from Hokkaido to Kyushu and Okinawa. Notably, these manifestations appeared earlier than the tsunami arrival time as a gravitational wave and were confirmed after a rapid increase in atmospheric pressure was observed. Then, the short-wave period component was predominant, and the wave height was amplified while an envelope was drawn. A 1.2 m tsunami was observed in Amami City at 23:55 JST on the January 15, and JMA announced a tsunami warning/advisory. The tsunami continued after that, and it took approximately one day to cancel the warning. In the future, for tsunamis caused by volcanic eruption activity, it will be necessary to analyze the tsunami component based on the sudden increase in atmospheric pressure known as air vibration in addition to components generated near the eruption to explain the progress of a tsunami. Confusion was also reported in various coastal areas of Japan due to the suddenness of tsunami information received at night in winter and the change in the JMA preliminary forecast. Although an evacuation order was issued by the government, the evacuation ratio was as low as a few percent, and there were reports of traffic congestion caused by evacuation using cars. Ultimately, no damage was reported due to the inundation of the tsunami, but limited damage did occur in the sea area. There were reports of fishing boats sinking and damage to aquaculture facilities; thus, future measures are needed for economic support of marine product facilities.

## References

[CR2] Asahi, S. (2022a). Tonga volcano eruption triggers tsunami warnings in Japan, Pacific islands. 16 January. https://www.asahi.com/ajw/articles/14522999

[CR3] Asahi, S. (2022b) 3/11 survivors evacuate to high ground following Tonga eruption. 17 January. https://www.asahi.com/ajw/articles/14523839

[CR4] Choi BH, Pelinovsky E, Kim KO, Lee JS (2003). Simulation of the trans-oceanic tsunami propagation due to the 1983 Krakatau volcanic eruption. Natural Hazards and Earth System Sciences.

[CR5] CNN. (2022). Tsunami advisories lifted in US after waves hit Tonga following volcanic eruption. https://edition.cnn.com/2022/01/15/asia/tsunami-warning-tonga-volcano-intl-hnk/index.html

[CR6] Harkrider D, Press F (1967). The Krakatoa air-sea waves: An example of pulse propagation in coupled systems. Geophysical Journal International.

[CR1] Japan Meteorological Agency (JMA). (2022). Tsunami Warning/Advisory and Tsunami Information Tsunami Information. https://www.data.jma.go.jp/svd/eqev/data/en/guide/tsunamiinfo.html

[CR7] Kato, H., Tanji, Y., Fujima, K., & Shigihara, Y. (2010). Study on Measures against Drifting of Cultivation Rafts by Tsunami (Report on the result of 2010 Chili Earthquake Tsunami). *Collection of articles published by the Japanese Institute of fisheries Infrastructure and Communities* (vol. 21, pp. 111–120) (**in Japanese with English abstract**)

[CR8] Kawamata, S., Imamura, F., & Shuto, N. (1992). Numerical simulation of the 1883 Krakatau tsunami. *Proceedings of 20th IAHR*, Tokyo.

[CR9] Kyodo. (2022). Japan sees tsunami but no major damage after Tonga volcano eruption. 16 January. https://english.kyodonews.net/news/2022/01/cf82db4e9f53-urgent-japans-pacific-coast-hit-by-tsunami-after-tonga-eruption.html

[CR10] Muhari A, Charvet I, Futami T, Suppasri A, Imamura F (2015). Assessment of tsunami hazard in port and its impact on marine vessels from tsunami model and observed damage data. Natural Hazards.

[CR11] Nomanbhoy N, Satake K (1995). Generation mechanism of tsunamis from the 1883 Krakatau eruption. Geophysical Research Letters.

[CR12] Pelinovsky, E., Choi, B. H., Tromkov, A. S, Didenkulova, I., & Kim, H.-S. (2005). Analysis of tide-guage records of the 1883 Krakatau tsunami, Case studies and Recent development. In K. Satake (Ed.) (pp 57–78). Springer.

[CR13] Press F, Harkrider D (1966). Air-sea waves from the explosion of Krakatoa. Science.

[CR14] Reuters. (2022). Significant tsunami damage feared in Tonga, communications still cut. 16 January. https://jp.reuters.com/article/us-tonga-volcano-idCAKBN2JQ001

[CR15] Suppasri A, Fukui K, Yamashita K, Leelawat N, Hiroyuki O, Imamura F (2018). Developing fragility functions for aquaculture rafts and eelgrass in the case of the 2011 Great East Japan tsunami. Natural Hazards and Earth System Sciences.

[CR16] Suppasri A, Muhari A, Futami T, Imamura F, Shuto N (2014). Loss functions of small marine vessels based on surveyed data and numerical simulation of the 2011 Great East Japan tsunami. Journal of Waterway, Port, Coastal and Ocean Engineering-ASCE.

[CR17] Symons GJ (1888). The eruption of Krakatoa and subsequent phenomena.

[CR18] The Gaurdian. (2022). Two drown in Peru as abnormally big waves from Tonga volcano hit coast. https://www.theguardian.com/world/2022/jan/17/two-drown-in-peru-as-abnormally-big-waves-from-tonga-volcano-hit-coast

[CR19] The Japan Times. (2022). Japan sees meter-high waves and tsunami warning after massive Tonga eruption. 16 January. https://www.japantimes.co.jp/news/2022/01/16/national/japan-tsunami-tonga-volcano/

[CR20] The Mainichi. (2022). Only 4% evacuated in northeast Japan area hit by tsunami after Tonga volcanic eruption. 27 January. https://mainichi.jp/english/articles/20220127/p2a/00m/0na/002000c

[CR21] Yokoyama I (1981). A geophysical interpretation of the 1883 Krakatau eruption. J. Volcanology and Geothermal Research.

